# Intensity of Grandparent Caregiving and Well-Being in a Cultural Context: A Systematic Review

**DOI:** 10.1093/geroni/igab046.1900

**Published:** 2021-12-17

**Authors:** Athena Chung Yin Chan, Sun-Kyung Lee, Jingchen Zhang, Jasmine Banegas, Scott Marsalis, Abigail Gewirtz

**Affiliations:** 1 University of Minnesota -- Twin Cities, St Paul, Minnesota, United States; 2 University of Minnesota -- Twin Cities, St. Paul, Minnesota, United States; 3 University of Minnesota -- Twin Cities, Minneapolis, Minnesota, United States; 4 University of Minnesota -- Twin Cities, St Paul., Minnesota, United States

## Abstract

With improved longevity and changes in family structure, grandparents are key resources in providing care for grandchildren. However, mixed findings indicate that multiple role engagement may enhance well-being or bring demands on grandparents raising grandchildren. Little is known about how the intensity of grandparent caregiving is associated with their well-being in different family contexts (i.e., structures, cultures/regions, and reasons of care). This systematic review examines the association between the intensity of grandparent caregiving and their well-being. Peer-reviewed articles published after 1990 were identified in five electronic databases. A keyword search was performed for keywords associated with: (a) grandparent caregivers raising grandchildren, and (2) well-being (i.e., physical, mental, cognitive, and life satisfaction). Only quantitative studies were included. Fifty-six articles from 28 countries/regions were included. Findings suggested that the well-being of grandparents is optimal when they provide caregiving of moderate intensity, with optimal amounts varying across sociocultural contexts. In Europe and Australia, providing supplementary care seems beneficial for grandparents’ well-being, especially supporting dual-earner families. In Asia, economic resources buffer the adverse effect of primary care on grandparents’ well-being. In the U.S., findings vary across ethnicity/race. White grandparents enjoy health benefits providing supplementary care with support from adult children. However, Hispanic grandparent caregivers in multigenerational households have better well-being than those in skipped-generation households, whereas Black custodial caregivers have better well-being than supplemental caregivers. Collectively, the intensity of grandparent caregiving and well-being is complicated by their roles in the family and cultural differences. This systematic review calls for culturally-tailored family interventions.

